# A Case of Paget-Schroetter Syndrome in a Young, Healthy Woman

**DOI:** 10.7759/cureus.89588

**Published:** 2025-08-07

**Authors:** Jane Ma, Patrick H Poquiz

**Affiliations:** 1 Internal Medicine, University of California Los Angeles, Los Angeles, USA

**Keywords:** effort-induced upper extremity deep vein thrombosis, paget-schroetter syndrome, thoracic outlet compression, upper extremity thrombosis, venous thoracic outlet syndrome

## Abstract

This report discusses a case of a 33-year-old healthy woman who presented with upper extremity swelling and pain, which she attributed to an injury sustained during her work as a professional dancer. Given her persistent symptoms, she was eventually referred to the emergency room for evaluation of possible thrombosis. She was found to have an elevated D-dimer, and a CT angiogram of the chest revealed narrowing of the bilateral subclavian veins suggestive of venous thoracic outlet syndrome (VTOS). A right upper extremity deep vein thrombus was subsequently confirmed on ultrasound imaging. The patient was started on anticoagulation, vascular surgery was consulted, and the patient was diagnosed with Paget-Schroetter syndrome. She underwent thrombolysis and venoplasty with symptomatic improvement. Decompressive surgery of the thoracic outlet was recommended to the patient as a means of definitive treatment to avoid recurrent thrombosis; however, the patient elected to defer at this time due to her active occupation. This case illustrates how this condition is diagnosed, the pathophysiology behind it, and the various treatment options available. It highlights the importance of shared decision-making between patient and physician in determining management that best fits the patient’s lifestyle and goals.

## Introduction

Paget-Schroetter syndrome (PSS) is a condition in which thrombosis occurs in the axillary or subclavian veins with compression of the subclavian vein at the thoracic outlet. Statistically, it is seen in younger patients and is more commonly seen in males in comparison to females [1], although there appears to be an increasing occurrence in women who participate in sports [2]. It occurs more commonly on the right side, possibly due to right-hand dominance [1]. Often, those who are diagnosed with PSS tend to be athletes who perform repetitive over-the-head arm movements, with injury to or compression of the axillary-subclavian vein leading to venous injury and thrombosis [3]. Patients can present with increased swelling, pain, and girth of the extremity. On occasion, venous collaterals may develop around the chest or shoulder. Workup can be initiated with an ultrasound to diagnose thrombosis. Computed tomography (CT) venography or magnetic resonance (MR) venography can also demonstrate thrombus in the venous system. Once diagnosed, thrombophilia labs such as D-dimer, protein C, protein S, factor V Leiden, prothrombin gene mutation, and antithrombin levels are also typically sent. PSS is first treated with anticoagulation, followed by options for thrombolysis or thrombectomy, and surgical decompression of the thoracic outlet.

## Case presentation

A 33-year-old healthy woman was experiencing right upper extremity swelling and pain for several days after what she thought was an injury sustained during a professional dance rehearsal. She reported repetitive swinging motions of her arms and, over the course of five days, developed increasing soreness and achiness, as well as numbness and tingling in the right hand, along with coolness of the extremity to touch. She initially presented to urgent care. However, given the progression of her symptoms, there was concern for thrombosis, and she was referred to the emergency room for evaluation. She denied shortness of breath, chest pain, or cough, and her review of systems was otherwise negative. She did not have any knowledge of a family history of thrombosis or any other blood disorders.

Upon arrival to the emergency room, she was afebrile with a temperature of 36.5°C, heart rate of 78 beats per minute (bpm), respiratory rate of 20 breaths per minute, blood pressure of 136/83 mmHg, and saturating 100% on room air. Her physical examination was notable only for diffuse mild right upper extremity edema with mild tenderness to palpation, and mild erythema noted on the right hand. Her initial laboratory findings showed a normocytic anemia with a hemoglobin of 11 g/dL; her complete metabolic panel was largely unremarkable, but she was noted to have an elevated D-dimer at 3.96 μg/mL. Initial labs upon presentation are shown in Table 1. Given the abnormal D-dimer, the emergency department ordered a CT angiogram of the chest, which was negative for pulmonary embolism, but noted to have narrowing of the bilateral subclavian veins at the thoracic outlet triangle, raising concern for venous thoracic outlet syndrome (VTOS). A Doppler ultrasound of the right upper extremity veins confirmed the presence of an acute deep vein thrombosis in the axillary, brachial, and subclavian veins. She was subsequently started on therapeutic enoxaparin, and vascular surgery was consulted.

**Table 1:** Laboratory results on initial presentation. WBC: white blood cell; Hgb: hemoglobin; ALT: alanine aminotransferase; AST: aspartate aminotransferase

Test	Result	Reference range
WBC	9.3 K/μL	4.1-11.1 K/μL
Hgb	11.0 g/dL	12.0-16.0 g/dL
Platelets	200 K/μL	150-400 K/μL
D-dimer	3.96 μg/mL FEU	<0.50 μg/mL FEU
Sodium	142 mmol/L	136-145 mmol/L
Potassium	3.6 mmol/L	3.5-5.1 mmol/L
Chloride	108 mmol/L	98-107 mmol/L
Carbon dioxide	26 mmol/L	20-31 mmol/L
Anion gap	8 mmol/L	-
Glucose	108 mg/dL	74-106 mg/dL
BUN	10 mg/dL	9-23 mg/dL
Creatinine	0.66 mg/dL	0.55-1.02 mg/dL
Albumin	4.5 g/dL	3.2-4.8 g/dL
Total protein	7.2 g/dL	5.7-8.2 g/dL
Calcium	9.4 mg/dL	8.7-10.4 mg/dL
Alkaline phosphatase	69 U/L	46-116 U/L
ALT	<7 U/L	10-49 U/L
AST	16 U/L	<33 U/L
Total bilirubin	0.2 mg/dL	0.2-1.0 mg/dL

Vascular surgery confirmed that the presence of thrombus was likely due to PSS in the setting of VTOS. Her profession as a dancer historically explains effort-induced thrombosis with compression of the subclavian vein at the thoracic outlet triangle. Given her active career, surgical interventions, including thrombolysis, thrombectomy, and debulking, were offered to her as opposed to conservative medical management with prolonged anticoagulation. The patient opted to proceed with thrombolysis and thrombectomy and delay surgical debulking given the recovery and rehabilitation efforts needed post-surgically.

**Figure 1:**
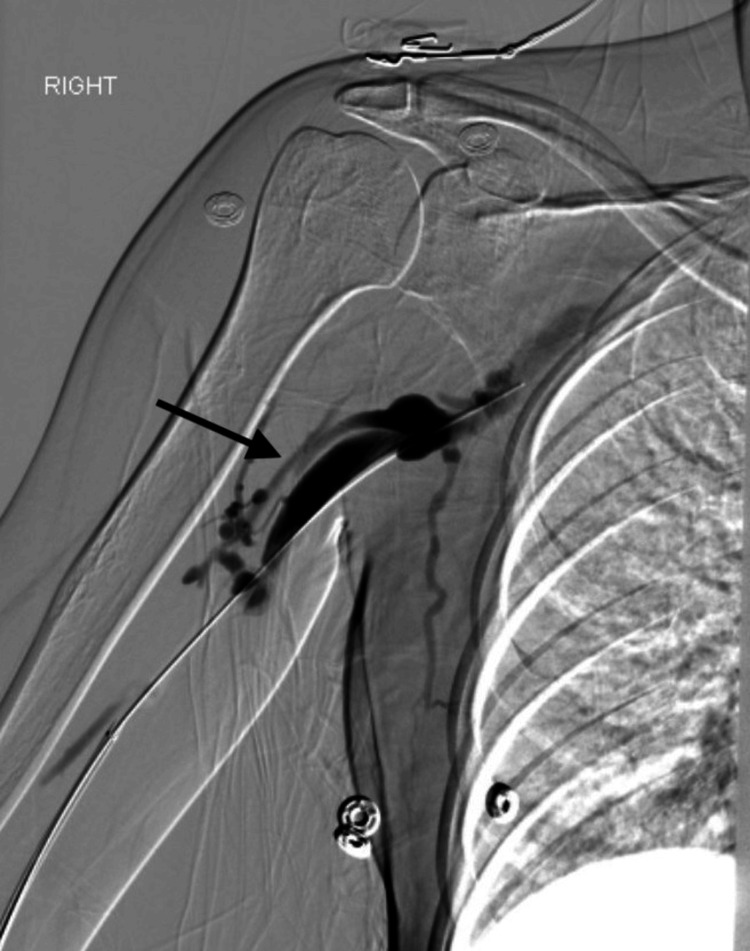
Initial venogram showing significant contrast build-up and subsequent decreased flow due to clot burden and thrombosis in the right subclavian vein (arrow).

Vascular surgery was performed on the right upper extremity, including a central venogram and multi-level intravascular ultrasound. With ultrasound, they were able to identify compression of the subclavian vein when the right upper extremity was passively moved with adduction and abduction. This confirmed the diagnosis of VTOS. Large thrombosis was noted in the right subclavian and axillary veins; thus, a lysis catheter was placed in the right arm, and the patient was initiated on thrombolysis therapy with heparin and alteplase infusions (Figure 1). She was transferred to the intensive care unit for close monitoring, given the high risk for bleeding.

**Figure 2:**
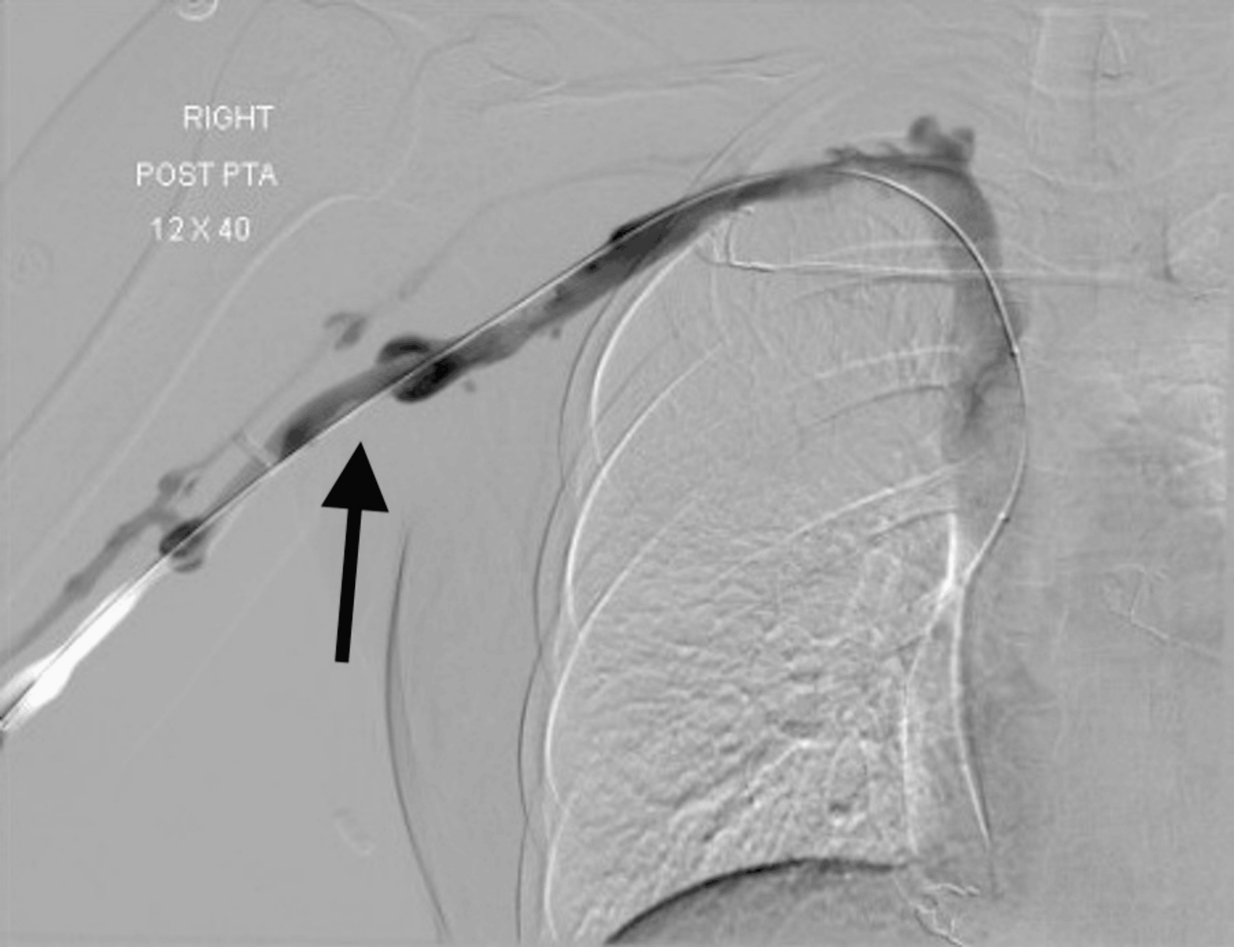
Venogram performed status post balloon venoplasty and catheter-directed thrombolysis of the subclavian and innominate veins (arrow), showing significant improvement in contrast flow.

The next day, the patient reported symptomatic improvement in right-hand swelling and soreness. The vascular surgery team took the patient back to the procedure room for a repeat venogram. Residual thrombus persisted; thus, she underwent a balloon venoplasty at the right subclavian and innominate vein junction and continued thrombolysis therapy via infusion. Approximately 10 h later, she returned to the procedure room for yet another venogram and underwent additional balloon venoplasty twice at the same segment to ultimately reduce the residual thrombus and stenosis to less than 10% (Figure 2). It was then that lysis therapy was terminated, and the catheter was discontinued.

She was observed an additional night in the ICU and did not sustain bleeding complications from thrombolysis therapy. She continued to endorse decreased swelling to her entire right upper extremity at the time of discharge. She was started on a loading dose of apixaban and was instructed to maintain therapeutic dosing for a minimum of six months. Her immediate follow-up was scheduled for four weeks post-hospitalization, with plans to repeat a venous ultrasound of the right upper extremity and scheduling of elective surgical debulking.

Vascular surgery team advised that decompression surgery is recommended within six months of diagnosis of VTOS; however, given her active career as a dancer, she opted to postpone surgical debulking, as this would likely mean interruption of her occupational schedule. She is compliant with her follow-up appointments and her medications as she continues to remain on anticoagulation with apixaban. Fortunately, at each surveillance imaging appointment, there has not been a recurrence of thrombus; however, vascular surgery continues to advise decompression at the patient’s earliest convenience.

## Discussion

The treatment for PSS is initiated with anticoagulation, typically with intravenous heparin or enoxaparin, and then transitioned to vitamin K antagonists or direct oral anticoagulants. If the patient presents within a two-week window, venogram and catheter-directed thrombolysis are considered to reduce clot burden, as it is considered to be most effective in that timeframe [4]. Mechanical catheter-directed thrombectomy can also be considered. There is no consensus as to what the optimal treatment regimen is. What is considered by many to be the definitive treatment of PSS is via decompression of the thoracic outlet. In the case of the above patient, vascular surgery outlined decompression as right scalenectomy, first rib resection, venal lysis, and percutaneous venoplasty. Various case studies note that catheter-directed thrombolysis and first rib resection result in improved outcomes and functional status, with thrombus and symptom resolution, and lower risk of complications such as recurrence of thrombus and post-thrombotic syndrome [4-6]. Yet other studies suggest that anticoagulation and catheter-directed thrombolysis alone are sufficient [7]. With respect to catheter-directed thrombolysis, it can provide benefit in the way of early clot lysis, shorter lysis time, and increased chance of success with initial treatment. Potential post-operative complications include post-operative pneumothorax, bleeding complications, and short-term neurologic complications [4]. More studies are likely needed to compare these treatment modalities and to ascertain what the optimal treatment regimen may be.

The timing of decompression after thrombolytic therapy is unclear; however, some studies suggest no statistical difference in symptom resolution in patients who have first rib resection in less than six weeks versus more than six weeks [8]. In terms of determining anticoagulation duration, a venogram is sometimes performed about two weeks after the decompression to help decide on the need for anticoagulation. An underlying hypercoagulable state or disorder may also affect the duration of anticoagulation. Potential complications of PSS include pulmonary embolism, recurrent thrombosis, or post-thrombotic syndrome. Treating patients with anticoagulation alone appears to increase the risk of persistent symptoms.

## Conclusions

This case highlights the importance of diagnosing PSS and initiating treatment as soon as possible, given its potential impact on functional outcomes and quality of life. Additionally, treatment and follow-up may need to be tailored to the needs and goals of each individual patient, as was done with our patient. Current guidelines in treating this syndrome are based on small case series and case studies, as well as consultant expert opinions, as there is no consensus on the optimal treatment of this condition. More prospective and large-scale studies, as well as randomized controlled trials, are needed to help evaluate the efficacy of thrombolysis and surgical treatment options.

## References

[1] Saleem T, Baril DT (2023). Paget-Schroetter syndrome. StatPearls [Internet].

[2] Ilhan E, Ture M, Yilmaz C, Arslan M (2009). Subclavian vein thrombosis extending into the internal jugular Vein: Paget-von Schroetter syndrome. J Clin Med Res.

[3] Shebel ND, Marin A (2006). Effort thrombosis (Paget-Schroetter syndrome) in active young adults: current concepts in diagnosis and treatment. J Vasc Nurs.

[4] Hoexum F, Hoebink M, Coveliers HM, Wisselink W, Jongkind V, Yeung KK (2023). Management of Paget-Schroetter syndrome: a systematic review and meta-analysis. Eur J Vasc Endovasc Surg.

[5] Karaolanis G, Antonopoulos CN, Koutsias SG (2021). A systematic review and meta-analysis for the management of Paget-Schroetter syndrome. J Vasc Surg Venous Lymphat Disord.

[6] Chun TT, O'Connell JB, Rigberg DA (2022). Preoperative thrombolysis is associated with improved vein patency and functional outcomes after first rib resection in acute Paget-Schroetter syndrome. J Vasc Surg.

[7] Silverberg D, Fish M, Lubetsky A, Rimon U, Raskin D, Greenberg G, Halak M (2021). Long-term outcome after nonsurgical management of Paget-Schroetter syndrome. J Vasc Surg Venous Lymphat Disord.

[8] Sangani V, Pokal M, Balla M, Gayam V, Konala VM (2021). Paget-Schroetter syndrome in a young female. J Investig Med High Impact Case Rep.

